# How to implement person-centred care and support for dementia in outpatient and home/community settings: Scoping review

**DOI:** 10.1186/s12913-022-07875-w

**Published:** 2022-04-22

**Authors:** Nidhi Marulappa, Natalie N. Anderson, Jennifer Bethell, Anne Bourbonnais, Fiona Kelly, Josephine McMurray, Heather L. Rogers, Isabelle Vedel, Anna R. Gagliardi

**Affiliations:** 1 Toronto General Hospital Research Institute, University Health Network, 200 Elizabeth Street, 13EN-228, Toronto, ON M5G2C4 Canada; 2 Toronto Rehabilitation Institute, University Health Network, 550 University Avenue, Toronto, ON M5G 2A2 Canada; 3 Faculty of Nursing, Université de Montréal, PO Box 6128, Montreal, QC H3C 3J7 Canada; 4 Division of Nursing, Queen Margaret University, Queen Margaret University Drive, Musselburgh, East Lothian EH21 6UU UK; 5 Lazaridis School of Business and Economics/Health Studies, Wilfrid Laurier University, 73 George Street, Brantford, ON N3T 3Y3 Canada; 6 Biocruces Bizkaia Health Research Institute and Ikerbasque Basque Foundation for Science, Bilbao, Spain, Plaza Cruces s/n, E-48903 Barakaldo, Spain; 7 Department of Family Medicine, McGill University, 5858 Côte-des-Neiges, Montreal, QC H3S 1Z1 Canada

**Keywords:** Neurocognitive disorder, Dementia, Person-centred care, Outpatient care, Home care, Implementation, Scoping review

## Abstract

**Background:**

Little prior research focused on person-centred care and support (PCCS) for dementia in home, community or outpatient care. We aimed to describe what constitutes PCCS, how to implement it, and considerations for women who comprise the majority of affected persons (with dementia, carers).

**Methods:**

We conducted a scoping review by searching multiple databases from 2000 inclusive to June 7, 2020. We extracted data on study characteristics and PCCS approaches, evaluation, determinants or the impact of strategies to implement PCCS. We used summary statistics to report data and interpreted findings with an existing person-centred care framework.

**Results:**

We included 22 studies with qualitative (55%) or quantitative/multiple methods design (45%) involving affected persons (50%), or healthcare workers (50%). Studies varied in how PCCS was conceptualized; 59% cited a PCC definition or framework. Affected persons and healthcare workers largely agreed on what constitutes PCCS (e.g. foster partnership, promote autonomy, support carers). In 4 studies that evaluated care, barriers of PCCS were reported at the affected person (e.g. family conflict), healthcare worker (e.g. lack of knowledge) and organizational (e.g. resource constraints) levels. Studies that evaluated strategies to implement PCCS approaches were largely targeted to healthcare workers, and showed that in-person inter-professional educational meetings yielded both perceived (e.g. improved engagement of affected persons) and observed (e.g. use of PCCS approaches) beneficial outcomes. Few studies reported results by gender or other intersectional factors, and none revealed if or how to tailor PCCS for women. This synthesis confirmed and elaborated the PCC framework, resulting in a Framework of PCCS for Dementia.

**Conclusion:**

Despite the paucity of research on PCCS for dementia, synthesis of knowledge from diverse studies into a Framework provides interim guidance for those planning or evaluating dementia services in outpatient, home or community settings. Further research is needed to elaborate the Framework, evaluate PCCS for dementia, explore determinants, and develop strategies to implement and scale-up PCCS approaches. Such studies should explore how to tailor PCCS needs and preferences based on input from persons with dementia, and by sex/gender and other intersectional factors such as ethnicity or culture.

**Supplementary Information:**

The online version contains supplementary material available at 10.1186/s12913-022-07875-w.

## Background

Dementia is expected to affect 152 million people by 2050, and is the second largest cause of disability for older persons and the seventh leading cause of death [1]. Dementia refers to mild, moderate or advanced cognitive impairment that affects memory, cognitive function, behaviour and ability to perform activities of daily living [2]. Alzheimer’s disease accounts for 60–80% of cases [2]. People with dementia have complex psychological, social and biomedical needs, which largely fall on caregivers (i.e. family/carers), negatively impacting caregiver employment, health and well-being [1, 2]. The cost imposed by dementia is approximately USD $818 billion per year globally, a considerable burden for healthcare systems and society at large [1]. To improve care and support for those affected by dementia, the World Health Assembly created a global dementia action plan in 2017 calling for research and innovation on risk reduction, diagnosis, treatment, and support for people with dementia [3], prompting the development of dementia strategies in many countries.

Person-centred care involves partnership with patients and family carers to tailor care to clinical needs, life circumstances and personal preferences; and offer knowledge, skills and access to supports that optimize quality of life [4, 5]. While person- rather than disease-centred dementia care is recognized as a worldwide priority [3, 6, 7], a scoping review (88 studies, 1998–2015) revealed little insight on how to implement it [8]. Instead, dementia research arbitrarily labelled “person-centred” has largely focused on diagnosis or clinical management, particularly in institutional settings [9]. Given that the majority of persons with dementia live at home, high quality outpatient care and support can reduce hospitalization or emergent care, and prevent or delay institutionalization [10, 11]. Research on outpatient primary care showed that patient-centredness of consultations decreased with increasing visit complexity [12], and specific to dementia, several person-centred care constraints (e.g. lack of time, low reimbursement, lack of interdisciplinary teams or links with community agencies) and unfavourable attitudes to person-centred care (e.g. belief that care and support should be provided elsewhere by community and social services) delayed detection of problems, and increased reliance on pharmacological rather than psychosocial management approaches [13, 14]. Similarly, research found that home or community support services (e.g. physical therapy, meal preparation) did not meet patient or caregiver needs because they were standardized rather than person-centred [10, 15–17].

Person-centred care is proven to enhance patient-important and clinical outcomes [18, 19] and widely advocated for those affected by dementia [3, 6, 7], yet person-centred dementia care and support appear to be lacking [12–14]. In particular, dementia disproportionately affects women. Two-thirds of persons with dementia are women, an escalating reality as older persons are increasingly women, the symptoms they live with are more severe, most caregivers of the nearly 70% of home-dwelling persons with dementia are wives or daughters, and more women than men are likely to be institutionalized [1, 20, 21]. This has led to calls for greater insight on gender issues of living with or caring for someone with dementia [20]. Such insight could be used to tailor care and support for women, who are not a homogeneous group, according to intersectional factors including but not limited to age, ethno-cultural background and socioeconomic status. The purpose of our research was to synthesize published research and generate insight on how to achieve person-centred dementia care and support (PCCS). The objectives were to identify: (1) what constitutes PCCS, (2) how to implement PCCS in outpatient or home−/community-based care including policies, programs, interventions or tools aimed at patients, carers or clinicians, (3) how to tailor PCCS for women given that two-thirds of persons with dementia are women as are most carers of persons with dementia [1, 20, 21], and to (4) generate a framework of PCCS for dementia.

## Methods

### Approach

We conducted a scoping review comprised of five steps: scoping, searching, screening, data extraction, and data analysis, and complied with standard methods and a reporting checklist specific to scoping reviews [22, 23]. Similar in rigour to a systematic review, we chose a scoping review because it accommodates a range of study designs and outcomes, establishes baseline knowledge on a given topic, and reveals gaps in knowledge that warrant ongoing primary research [24]. The review question was how to implement PCCS. The research team, included clinicians (primary care, nursing) and health services researchers with expertise in the topics of dementia, person-centred care, patient engagement and women’s health, and the methods of qualitative and quantitative research, and syntheses; who contributed to multiple components: conceptualization, study design, eligibility criteria, review and interpretation of data, and report preparation. We did not register a protocol as scoping reviews are not accepted by PROSPERO. The University Health Network Research Ethics Board did not require approval as data were publicly available, and we did not register a protocol.

### Scoping

To familiarize ourselves with potentially relevant literature, we conducted an exploratory search in MEDLINE using medical subject headings (MeSH): dementia or Alzheimer’s disease AND patient-centered care. After reviewing search results and identifying examples of relevant studies, we generated eligibility criteria based on a PICO (participants, issue, context, outcomes) framework and planned a targeted, comprehensive search strategy.

### Eligibility

Detailed eligibility criteria are described in Additional File 1. In brief, *participants* were community-dwelling persons with diagnosed dementia or caring for a person with dementia, or healthcare workers providing outpatient, home- or community-based (i.e. day centres) care or support to such persons. The *issue* of interest was person-centred care, defined for this study based on a six-domain framework by McCormack et al. as a multi-dimensional approach to care that fosters a healing relationship, exchanges information, addresses emotions or concerns, manages uncertainty, shares decision-making, and enables self-management [25]. Though not specific to dementia, we chose this framework because it was robustly developed with input from patients, carers and clinicians, and with 28 items in six domains, offers a thorough description of person-centred care [25]. Throughout this manuscript we refer to person-centred care, which acknowledges the longstanding concepts of personhood and holistic care in the dementia context [8, 9]. However, given the interchangeable use of terms for person-centred care, we used an inclusive approach where person-centred care could be referred to as patient- or person-centred care, family-centred care or a synonymous term. In prior research we elaborated on approaches within those domains [26, 27]. For example, approaches to foster a healing relationship included establish rapport (engage in brief, friendly conversation prior to clinical discussion) and assume a non-judgmental attitude (speak in a respectful manner). In addition to person-centred care approaches such as these, we were also interested in strategies aimed at patients/carers or healthcare workers to implement (i.e. promote/support) use of person-centred care approaches including policies, programs, interventions or tools. *Context* referred to studies exploring or describing what patients, carers or healthcare workers view as person-centred care approaches; determinants influencing the use or impact of person-centred care approaches (enablers, barriers), or the impact of strategies (policies, programs, interventions, tools) to promote or support PCC approaches targeted to patients, carers or healthcare workers. Study design referred to empirical research with explicit methods of data collection and analysis including qualitative (e.g. interviews, focus groups), quantitative (e.g. questionnaires, retrospective or prospective cohort studies, trials) or multiple/ mixed methods research published in English language. *Outcomes or impacts* included but were not limited to awareness, knowledge, practice or impact of person-centred care approaches, or determinants of use or impact. To focus on home- or community-based PCCS, we excluded studies if participants were trainees, the context was long term or palliative care, person-centred care referred to clinical care/management. We also excluded protocols, abstracts, editorials, letters, commentaries, clinical case studies, or clinical guidelines. Reviews were not eligible but we screened references for eligible studies.

### Searching

ARG, who has medical librarian training, developed a search strategy that complied with the Peer Review of Electronic Search Strategy reporting criteria (Additional File 2) [28]. In our prior experience, a search employing the MeSH term “patient-centered care” generates a very large number of results including a diffuse range of un-related topics [29]. To better target studies of PCC approaches, our search strategy combined the MeSH term “patient-centered care” with keywords for synonymous terms. NNA searched MEDLINE, EMBASE, CINAHL, SCOPUS, the Cochrane Library, and Joanna Briggs Institute Database of Systematic Reviews from January 1, 2000 to June 7, 2020. We chose 2000 as the start date to coincide with the emergence of widespread advocacy for person-centred care [30]. We exported search results to Excel for screening.

### Screening

To pilot test screening, NM, NNA and ARG independently screened titles and abstracts for the first 50 search results, then compared and discussed discrepancies, and how to interpret and apply screening criteria. NM screened remaining titles/abstracts in duplicate with NNA, and they resolved uncertainty or discrepancy with ARG through discussion. NM retrieved and screened full-text articles concurrent with data extraction, and NNA or ARG prospectively resolved uncertainties as they arose.

### Data extraction and analysis

We extracted data on study characteristics (author, publication year, country, objective, research design, person-centred care model or theory, women sub-analyses) and person-centred care approaches, evaluations to assess person-centred care, determinants (enablers/barriers), or the impact of strategies to promote or support person-centred care. We described strategies using the Workgroup for Intervention Development and Evaluation Research reporting framework: content, format, delivery, timing and personnel [31]. As a pilot test, NM, NNA and ARG independently extracted data from three articles, then compared and discussed results to clarify what to extract and how. NM subsequently extracted all data with assistance from NNA, and ARG independently reviewed all data. We tabulated aspects of person-centred care extracted from included studies based on McCormack’s person-centred care framework [25We used summary statistics to describe study characteristics, the number of studies by type of participant or demographics (e.g. women), and the number and type of person-centred care domains addressed in studies. We used text to compile unique enablers and barriers, described the characteristics and impact of strategies to promote or support person-centred care approaches, and noted considerations specific to women with dementia or women carers of persons with dementia. We transformed all findings, including person-centred care concepts, enablers, barriers and recommendations into PCCS strategies, mapped them to McCormack’s person-centred care framework [25], and noted additional unique person-centred care elements identified in included studies.

## Results

### Studies included

**Fig. 1:**
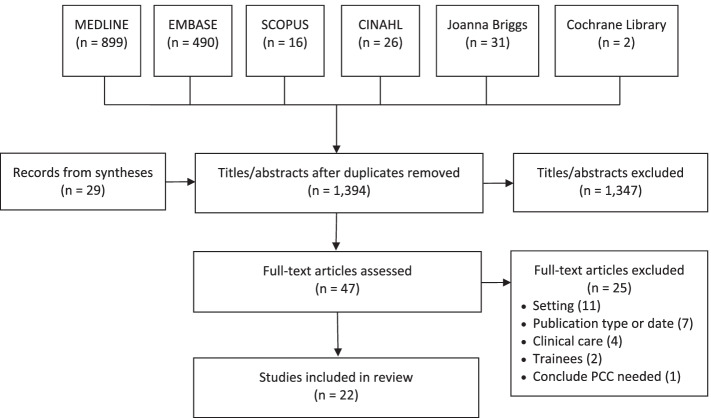
PRISMA diagram

A total of 1394 unique records were identified by searches and screening of review references, and 1347 were excluded by screening titles/abstracts. Among 47 full-text articles screened, 25 were excluded due to setting (11), publication type or date (7), topic was clinical care (4), participants were trainees (2) or the study only concluded that PCC was needed (1). A total of 22 studies were included for review (Fig. 1). Data extracted from included studies are available in Additional File 3 [32–52].

### Study characteristics

Studies were largely conducted in the United Kingdom (9,40.9%), United States (7, 31.8%) followed by Sweden (3, 13.6%) and one (4.5%) each in Canada, China and the Netherlands. Twelve (54.5%) studies employed qualitative methods including interviews (5), focus groups (5) or both (2). Eight (36.4%) studies employed quantitative methods including cohort study (5), randomized controlled trial (2), and survey (1). Two (9.1%) studies employed multiple methods: one a survey and focus groups, the other a randomized controlled trial and focus groups. With respect to setting, 11 (50.0%) studies addressed outpatient care (including primary care), and 11 (50.0%) addressed home or community day care. Ten (45.5%) studies addressed support, 7 (31.8%) addressed care, and 5 (22.7%) overall management including both care and support. Regarding participants, 1 (4.5%) included only persons with dementia, 7 (31.8%) both persons with dementia and carers, 3 (13.6%) carers only, 9 (40.9%) healthcare workers only and 2 (9.1%) on both carers and healthcare workers. Ten (45.5%) studies specified dementia stage: 5 mild cognitive impairment, 2 mild dementia, 2 both mild and moderate dementia, and 1 moderate dementia.

### PCC concepts

While all studies mentioned person-centred care concepts in their background or rationale, 13 (59.1%) studies explicitly referred to a person-centred care definition or framework, and most often this was Kitwood’s philosophy of personhood (6, 46.2%). When describing person-centred care, studies most often referred to a holistic approach involving partnership between healthcare workers and persons with dementia and carers, ensuring dignity and respect, recognizing the person’s life and current abilities, tailoring care to individual needs and preferences, optimizing independence by providing information and sharing decisions rather than taking over, and engaging in meaningful activity. Table 1 summarizes person-centred care concepts measured or generated by studies mapped to the McCormack person-centred care framework [26]. One study did not address any person-centred care domains because it focused on organizational enablers and barriers to implementing PCC [48]. The remaining 21 studies featured a median of 4 of 6 possible person-centred care domains (range 4 to 6). Most often, studies included the domains of address emotions (21, 100.0%) and exchange information (19, 90.5%). Least often, studies included the domains of enable self-care (9, 42.9%) and manage uncertainty (7, 33.3%).

**Table 1:** Person-centred care domains in included studies

Study	Person-centred care domains (n,% of 21 studies)						Total domains (n)
	Foster the relationship	Exchange information	Address emotions	Manage uncertainty	Share decisions	Enable self-care	
Berglund 2019 [53]	+	+	+	+	+	+	6
Hancox 2019 [32]	+	+	+		+	+	5
Ihara 2019 [33]		+	+		+		3
Hung 2018 [34]	+	+	+	+	+		5
Hall 2018 [35]	+	+	+	+	+		5
Jennings 2018 [36]	+	+	+			+	4
Chung 2017 [37]	+	+	+		+	+	5
Guan 2017 [38]		+	+	+	+		4
Wang 2017 [39]		+	+				2
Johansson 2017 [40]		+	+		+	+	4
Han 2016 [41]	+	+	+		+		4
Gaugler 2015 [42]	+	+	+	+	+		5
Edwards 2015 [43]	+	+	+				3
Smythe 2015 [44]	+	+	+	+			4
Edwards 2014 [45]		+	+	+		+	4
Lerner 2014 [46]	+	+	+	+	+		5
McClendon 2013 [47]	+		+		+		3
Kirkley 2011 [48]							0
Robinson 2010 [49]	+	+	+		+	+	5
Vernooji-Dassen 2010 [50]	+	+	+		+		4
Zaleta 2010 [51]	+		+			+	3
Ericson 2001 [52]	+	+	+		+	+	5
Total	14 (66.7)	19 (90.5)	21 (100.0)	7 (33.3)	15 (71.4)	9 (42.9)	median 4 range 4–6

### Identification of person-centred care approaches

Seven (31.8%) studies explored what constitutes person-centred care in community, home or outpatient settings. One study involving persons with dementia and carers generated a framework of 41 person-centred care approaches in five domains: medical care, physical quality of life, social and emotional quality of life, access to services and supports, and caregiver support [36]. That same study [36], plus two studies involving carers [37, 47], two studies involving healthcare workers [40, 44], and one study involving both [52] yielded common themes. Person-centred care approaches across these studies included: pay attention to verbal and behavioural cues to understand the affected person’s needs, promote autonomy and independence by engaging them in meaningful activity (e.g. family functions, planning and preparing meals), respect their abilities in a non-judgemental manner by practising kindness and patience, and create stability through routines and continuity. Healthcare workers emphasized working in partnership with persons with dementia and carers to enable decision-making rather than taking over, and the need to support carers, who said that constantly setting goals, gauging situations, making adjustments and negotiating through trial-and-error was stressful.

Carers and healthcare workers differed on one aspect. Carers were motivated to keep their affected family member at home because they were attuned to needs and preferences through intimate knowledge of the person, and were therefore best able to optimize care and support. Carers thought these essential qualities could not be acquired by healthcare workers through training. While healthcare workers agreed that home was the ideal environment, they believed it was not always possible. Healthcare workers believed that they could be equipped to provide person-centred care through training, and via continuity of one or a few healthcare workers who would develop insight to a person’s needs by getting to know them.

One additional study involving persons with dementia, carers and healthcare workers revealed four elements of person-centred diagnosis of dementia in primary care: reframing dementia as cognitive decline, thus allowing time for the affected person and carer to adjust to the diagnosis; paying attention to cues other than memory loss (e.g. behaviours, challenges) for recognizing dementia; engaging the entire primary care team (i.e. administrative or clerical staff) in identifying signs of dementia; being aware of available care and support services in the community and secondary care [45].

### Evaluation of PCC experience

Four (18.2%) studies assessed care and found it was not person-centred. One study interviewed persons with dementia and carers about physiotherapy [35]. Participants said that physiotherapists did not: look beyond dementia to get to know the affected person, tailor exercises or discuss how to adapt the exercise to overcome dementia-related difficulties (e.g. short routine), or clearly communicate the plan of treatment such that they felt confused and abandoned when it ceased. Three studies employed observation of recorded consultations between persons with dementia, carers and physicians or genetic counselors when dementia risk or diagnosis was disclosed [38, 46, 51]. Genetic counselors and physicians contributed the majority of utterances, which largely focused on biomedical information, providing lifestyle information and checking for understanding, and less often on expressing empathy or reassurance. Compared with such sessions, in those categorized as person-centred, healthcare workers gave less biomedical information, asked more psychosocial questions, and made more effort to build partnerships, and affected persons and carers contributed a greater proportion of utterances.

### PCC enablers and barriers

Table 2 summarizes enablers and barriers of person-centred dementia care and support reported in four (18.2%) studies [32, 44, 48, 50]. At the patient/carer level, enablers included practical strategies (memory aids, daily routine) and perceived value (tangible benefits, positive past experience), while barriers included lack of practical or emotional support from carer, reluctance to be helped, family conflict, and geographic or social distance between children and affected parents. At the healthcare worker level, individual enablers included mutual support from colleagues, job satisfaction and experiential learning through exposure to persons affected by dementia; and service enablers included awareness of family problems, following the family’s lead, maintaining a neutral disposition and creating a safe environment in which to offer support. Healthcare worker barriers included variable knowledge and understanding about person-centred care, negative attitudes about dementia, and perceived lack of control or time, and perceived low status within the organization. Organizational enablers included positive leadership style and support for staff, risk management, opinion leaders who championed and modeled person-centred care, and policy documents that promoted person-centred care. Organizational barriers included resource constraints, inadequate staffing and a stressful environment.

**Table 2:** Determinants of person-centred dementia care and support

Level	Enablers	Barriers
Patient or Carer	• Developing daily routine • Perceived or tangible benefits • Memory aids • Positive past experience	• Lack of practical or emotional support from their carer to routinize activity • Reluctance to be helped • Family conflict • Children geographically or socially distant from affected parent • Children feeling like unwanted intruder
Healthcare worker	• Mutual support from colleague • Job satisfaction • Experiential learning • Awareness of family dynamic problems • Maintaining neutral disposition • Following family lead • Creating a safe environment in which to offer help	• Variable knowledge/understanding of PCC • Attitudes about dementia • Perceived lack of control/time • Perceived low status within organization
Organization	• Leadership style that promotes PCCS • How managers support and value staff • Risk management • Opinion leaders who advocate and model PCCS • PCC integrated in policy documents	• Inadequate staffing • Resource constraints • Pressurized environment

### Strategies to implement PCC approaches

Table 3 summarizes the characteristics of strategies to promote or support the implementation of person-centred care approaches reported in 8 (36.4%) studies. Five studies targeted at healthcare workers used in-person educational meetings involving both didactic and interactive components [34, 39, 43, 49, 53]. Meetings ranged from single one-hour sessions to a three-day meeting, and all but one that involved psychiatrists [50] were interdisciplinary. All studies reported positive impacts. Based on qualitative studies, perceived impacts included beneficial patient health and outcomes (e.g. improved health and nutritional status, health problems detected earlier, referral for screening or to memory clinics, tailored support, reduced use of primary and hospital care, delayed institutionalization, patients and carers more engaged in decision-making) and benefits for healthcare workers (deeper insight and understanding of patient behaviour and needs, more compassion for patients as persons rather than symptoms to be managed, appreciation for the complexity of dementia care, enhanced team collaboration, greater job satisfaction). By instrument or survey data, educational meetings improved knowledge about, attitude to and use of person-centred care approaches to dementia (e.g. structure of consultations, communication techniques); and awareness of behavioural and functional symptoms, availability of support services and what constitutes person-centred care. Healthcare workers said that education was useful, would have a positive impact on their ability to provide dementia care, and in particular they valued communication techniques.

**Table 3:** Characteristics of strategies to promote or support the implementation of person-centred care approaches

Study	Goal (Research Design)	Intervention Design				
		Content	Format	Delivery	Timing	Personnel/Participants
Berglund 2019 [53]	Evaluate an educational program aimed at healthcare workers on how to provide person-centred home care (Qualitative – focus groups with 42 healthcare workers)	Session 1 - Dementia disease, associated disabilities, how to deal with problematic situations when delivering home care Session 2 - Psychiatric nursing, building a relationship, using conversation, adding emotions Session 3 - Models of care to support self-identity Session 4 - PCC: how to tailor and provide individualized care	Didactic and interactive with presentations, case studies and group discussions	In-person	Delivered over 4 to 6 months 4 sessions ranging from 30 min to 4 h	2 nurse experts in dementia care Care assistants, home care officers, registered nurses, physiotherapists, occupational therapists, and care managers
Ihara 2019 [33]	Evaluate the impact of a person-centred day care music listening intervention on mood, agitation, and social engagement for persons with dementia (Before-after cohort study of 31 persons compared with control group of 20 persons)	Personalized music playlist was developed by asking caregivers about the person’s favorite music or by playing different songs to gauge person’s reaction. Persons could listen to the same songs repeatedly or choose to listen to a variety of songs. Researchers shuffled the order of songs in each session	Person given headphones and iPod to listen to the personalized music playlist in a room with 7–10 others and closed door to minimize distractions	In-person	Single 1-h session: 20 min observation, 20 min music, 20 min post-observation	3 researchers, 5 graduate and undergraduate students
Hung 2019 [34]	Evaluate an educational program aimed at healthcare workers on how to provide person-centred outpatient dementia care (Multiple methods – survey, focus groups with 310 interdisciplinary healthcare workers)	Module 1 – PCC principles Module 2 - Common brain changes Module 3 – Communication and interpersonal strategies Module 4 – Self-protective skills and techniques	Didactic, small group learning exercises, story sharing, video vignettes, group reflections, and role-play	In-person	Single 1-day workshop, 12 people per workshop	Educators (number and characteristics not reported)
Wang 2017 [39]	Evaluate the impact of a train-the-trainer education a program for primary care professionals on dementia knowledge and attitudes, and person-centred outpatient care delivery (randomized controlled trial of 170 physicians and nurses + focus groups with 30 non-specified healthcare workers)	Enhancing early diagnosis of and responding to dementia in primary care, translating knowledge into practice	Modules included pre-reading, short lecture and interactive case study discussion; learning resources included a workbook and 4 DVD’s. Project team (lead nurse + 9 physicians and nurses) provided ongoing support for trainers through email, telephone and site visits	In-person	Trainer sessions: 3-day workshop of 20 h total comprised of 10 modules Delivery to peers: Weekly in-service education (number of weeks, hours not specified). Learners also completed self-study of required readings.	Trainers were 1 nurse and 1 physician from each intervention site
Han 2016 [41]	Explore how a social visit program offers person-centred support to persons with dementia and carers (qualitative – interviews with 5 carers)	Medical students were exposed to lectures on dementia fundamentals and communication skills for interacting with aging and cognitively impaired adults. They also took part in lunch meetings to share experiences with each other and program staff Students engaged persons with dementia (and sometimes carers) in social or cultural activity such as dinner or visiting a museum	Students received didactic (3-h lecture, monthly lunch-time speaker series), interactive (discussion with peers and program staff) and experiential training (reflection on learning) Students were paired with individuals based on shared interests and geographic proximity	In-person	Monthly meeting for minimum of four hours	First-year medical students received training in interaction with persons with dementia; persons with dementia were involved in social activity by medical students
Gaugler 2015 [42]	Evaluate the impact of an online educational program on carer knowledge of person-centred approaches to use in the home (Before-after cohort – survey of 41 carers)	3 modules: 1/ understanding memory loss Defines cognitive decline and explores impact on performance of activities of daily living 2/ living with dementia Strategies to help individuals with dementia function independently and safely and identifies tools for family caregiver stress management 3/approaches to manage behavioural problems	Videos offer vignettes and interviews with persons with dementia, family carers, professionals and national experts	Online	Three 1-h modules: 1/ 7 screens, 17 videos 2/ 18 screens, 4 videos 3/ 11 screens, 18 videos	Modules put together by a 14-person national expert panel comprising clinical and scientific experts in family caregiving
Edwards 2015 [43]	Evaluate the impact of an educational intervention to promote person-centred outpatient primary care for persons with cognitive decline (Before-after cohort – survey of 94 physicians, nurses and clerical staff)	Factual information and extracts from interviews with patients and carers to depict person-centred approaches to dementia and case examples; Introduction to dementia and the subtypes plus a case for early diagnosis; introduction to the concept of person-centred approaches to dementia based on extracts from interviews with people with dementia and their caregivers which cover: living with dementia; stigma and attitudes; early diagnosis; and seeking a diagnosis, along with seven case examples of people with cognitive decline presenting for consultation in primary care; the final section summarized the earlier sections and also included information on making referrals to memory services	Didactic and interactive; package included PowerPoint presentation, handbook of slides, 4 case examples for group discussion, training manual with detailed guidelines for delivery	In-person	Single 1-h session over a lunch time meeting	Developed by a service user, carer, researcher, consultant psychiatrist, academic GP and a consultant clinical psychologist, all with experience and interest in dementia. Delivered by one of the researchers
Robinson 2010 [49]	Evaluate the impact of an educational intervention to promote person-centred outpatient care by old age psychiatrists (Survey of 40 psychiatrists)	Theoretical aspects of person-centered care, facilitators and barriers, communication skills and approaches, all aimed to develop a therapeutic alliance, facilitated shared responsibility, promote patient autonomy, exploring patient experience and promoting quality of life	Didactic presentation, full group discussion, small group role-playing and self-reflection; 24 video clips showing demonstrating skills and approaches, and how to structure consultations	In-person	Single session of half day in length	2 or 3 of the study authors facilitated each workshop

Three studies targeted at persons with dementia or carers also reported positive impacts. One study evaluated an educational strategy targeted at carers. Based on a survey of carers, three one-hour online modules (text, videos) featuring vignettes and interviews with affected persons and carers increased knowledge and confidence in caregiving skills, and they appreciated the flexibility of online delivery of the educational program [42]. Two studies evaluated personalized strategies to implement meaningful activities. Based on third-party observation of persons with dementia, benefits of a single one-hour personalized music program improved patient mood (smiling, joy, alertness, relaxed, calm) and social engagement (eye contact, eye movement, talking), and decreased sleeping both during and directly after the session [33]. Interviews with carers revealed the benefits of a monthly four-hour social visit program (e.g. dinner, museum visit) from first-year medical students who were exposed to didactic and interactive educational meetings on dementia. Carers were pleased that their spouse enjoyed the program, it provided them with respite, and they also enjoyed participating in social activity. Carers said that persons with dementia benefited from an outlet to socialize with someone other than family, which provided them with social and intellectual stimuli [41].

### Person-centred care for women affected by dementia

Few (4, 18.2%) studies reported sub-analyses by sex/gender or other intersectional factors. In a survey of 148 carers (62% women) to identify what constitutes person-centred carer approaches, women carers were more likely to provide person-centred care at home compared with men [47]. In two studies involving observation of consultations to assess if disclosure of a dementia diagnosis was person-centred, among 262 (69.8% women, mean age 58.3 years, range 33–86 years) and 54 (61.1% women, mean age 74.1 years, range 58–91 years) persons, respectively, neither gender nor age were associated with communication patterns [46, 51]. In one study involving interviews with 20 persons with dementia (20% women) on enablers and barriers of adherence to a home-based facilitated activity program, eight participants had low adherence (3 women, 5 men) [32]. A variety of determinants influenced adherence, but sub-analyses by gender were not reported.

### Framework of person-centred care and support for dementia

Table 4 shows how the McCormack person-centred care framework [25] was confirmed and elaborated into the Framework of Person-Centred Care and Support for Dementia. Study findings confirmed the relevance of McCormack’s PCC domains and elements in the context of home, community/day and outpatient dementia care and support, and elaborated on that framework by more explicitly emphasizing some elements and contributing new elements. For example, details that corresponded with existing components of the domain of foster a dealing relationship that emerged included: discuss roles and responsibilities, communicate with honesty and openness, foster trust, express caring and sympathy, and build rapport. In addition to these elements, included studies also emphasized the need for partnership with persons with dementia and their carers to optimize the ability of healthcare professionals to offer and adjust support as needed throughout the disease trajectory. With respect to manage uncertainty, in addition to several practices that corresponded to the existing conceptual principles of this domain (e.g. raise and discuss known uncertainties, explore and assess uncertainties held by affected persons), included studies also highlighted the need to create stability through routines and continuity, which is particularly relevant to managing behavioural and psychosocial aspects of dementia.

**Table 4:** Framework of person-centred care and support for dementia

Domain	Elements	From this study
Foster a healing relationship	• Discuss roles and responsibilities • Communicate with honesty and openness • Foster trust in healthcare worker competence • Express caring and empathy • Build rapport	• Emphasize partnership • Ensure dignity and respect
Exchange information	• Explore needs and preferences • All parties share information • Provide/refer to additional information • Assess and facilitate understanding	• Recognize the person’s life and current abilities through discussion, and verbal and behavioural cues • Allow time for questions
Address emotions	• Explore and identify emotions • Assess anxiety or depression • Validate emotions • Express empathy or reassurance • Provide help to deal with emotions	• Reframe dementia as cognitive decline upon initial diagnosis to lessen impact • Address psychosocial issues in addition to biomedical
Manage uncertainty	• Raise and discuss uncertainties in prognosis, management or outcomes • Explore and assess other uncertainties • Use problem-focused (behavioural) management strategies • Use emotion-focused (affective) management strategies	• Create stability through routines and continuity
Share decisions	• Raise and discuss care or support options • Discuss decision process, and needs/support • Prepare persons/carers for deliberation and decisions • Jointly make and implement decisions and action plan • Assess decision quality and choices	• Tailor care and support to individual needs and preferences • Address or mitigate family conflict
Enable self-management	• Describe the follow-up process • Provide information and training on self-care and self-monitoring • Share guidance on how to prioritize and plan self-care • Offer practical advice and support to implement self-care • Assess skills, self-care and progress	• Optimize independence • Engage persons in meaningful activity • Support carers • Provide information about available home or community support/services

## Discussion

We aimed to describe what constitutes PCCS, how to implement it, and considerations for women who comprise the majority of affected persons – those with dementia and carers of persons with dementia. Regarding objective one, few studies were eligible, few explicitly referred to a person-centred care definition or framework, and studies varied in the person-centred care domains addressed. With respect to objective two, the few studies that assessed care or support found it was not person-centred; few studies explored enablers or barriers; and few studies evaluated the impact of strategies to implement PCCS. Objective three was not well-addressed because few studies reported results by gender or other intersectional factors, and none revealed if or how to tailor PCCS for women. Still, by synthesizing available research, we generated concrete insight and guidance including a framework of PCCS for dementia, multi-level enablers and barriers of PCCS for dementia, the characteristics of strategies to promote PCCS, and multiple remaining gaps in knowledge that warrant ongoing research.

This review contributes to a growing body of knowledge on PCCS for dementia. A number of existing articles on the topic of person-centred dementia care were excluded from this review. Some were discussion papers, for instance, anecdotal discussions of the confusion caused by variable terminology and lack of insight on how to achieve PCCS for dementia [54] or conceptual discussions related to the rising attention given to “centredness” [55]. Other papers focused on only one aspect of PCCS such as empathic communication [56], referred to implementing person-centred care but instead addressed clinical care [57, 58], or focused on hospital [59] or institutional care [9, 57, 60]. Other research has also explored the educational needs of trainees such as nursing assistants [61]. Therefore, this review offers a unique contribution to the literature in that it was based on a comprehensive framework inclusive of multiple person-centred care domains; focused on characterizing what constitutes PCCS in the home, community or outpatient setting; and described strategies used to implement PCCS in that setting.

One tangible output of this work was a framework of PCCS for dementia. While conceptualization of PCCS varied across studies, compilation of perspectives, enablers, barriers and recommendations for PCCS confirmed domains and elements in the existing McCormack person-centred care framework [25], and elaborated that framework with additional items relevant to dementia in home, community or outpatient settings. Notably, patients/carers and healthcare workers agreed on the majority of elements, further highlighting the relevance of the PCCS Framework we generated. Most studies referred to the person-centred care domains of address emotions and exchange information, reflecting the need to discuss and manage both the clinical and psycho-social impact of dementia. However, many studies did not address other person-centred care domains such as manage uncertaintyshare decisions and enable self-care, which are also highly germane to dementia, so this PCCS Framework represents a starting point, and we and other researchers can continue to refine it through ongoing research. Others, including Kitwood and Brooker, conceptualized PCCS in the dementia context, but their frameworks situated the person in the context of relationship and social being, and emphasized the philosophical approach of personhood, meaning respecting persons with dementia as individuals with unique needs and engaging them in their own care [62]. Others have also elaborated on the idea of personhood and what it means, and the need to acknowledge persons and their personality in dementia care and support [63, 64]. In contrast, the PCCS Framework we generated provides specific strategies that can be applied at the point of care. Healthcare workers, including clinicians and managers or staff of services that provide home or community care and support, can also refer to the framework as either the basis for strategic planning, or for evaluating and improving their services.

Few strategies to implement PCCS approaches were evaluated, and they largely focused on in-person interdisciplinary educational meetings for healthcare workers. While all intervention studies highlighted perceived, self-reported positive impacts on carer knowledge and confidence, and healthcare worker knowledge, attitude and skills, few studies objectively measured improvements or the impact on health and use of healthcare services among persons with dementia. Therefore, future research should more definitively evaluate the impact of educational meetings for healthcare workers, which appears to be promising in terms of improving and supporting PCCS. Other strategies also warrant investigation. For example, one study revealed that an enabler was opinion leaders who championed or modeled person-centred care [48]. The role of champions as positive influences on the implementation of healthcare interventions is well-established. A Cochrane systematic review showed that opinion leaders, credible and trustworthy individuals who disseminate and implement evidence-based practice through informal or formal mechanisms, improved quality of care [63]. Despite numerous patient/carer and organizational barriers of PCCS identified by included studies, few studies evaluated strategies targeted to persons with dementia or carers, and none evaluated organizational strategies. Thus, further research is needed to first identify and design strategies that match barriers, and then evaluate the impact of patient/carer and organizational strategies on a range of potential outcomes at various levels. Such research is likely best done in partnership with affected persons, which has been shown to be both feasible and fruitful [65, 66].

Despite recognition of the need for greater insight on gender issues of living with or caring for someone with dementia [20], this review identified a paucity of research that focused on PCCS for women or reported sub-analyses by sex/gender or other intersectional factors. This represents a critical gap in knowledge given that the majority of persons with dementia and carers are women [1, 20, 21]. The paucity of guidance on PCCS for women is particularly concerning given that analysis of national dementia strategies in 29 countries found they did not address sex or gender issues [67]. Recognizing that women are not a homogenous group, ongoing primary research is urgently needed to explore PCCS experiences and recommendations among diverse women, and to evaluate strategies for implementing PCCS that can be tailored to their individual needs and preferences.

Two additional issues also warrant further research. One, only four studies assessed PCCS, and found that physiotherapy support and disclosure of a dementia diagnosis were not person-centred, and only four studies explored enablers and barriers, knowledge essential to effectively designing and tailoring strategies to implement PCCS. Thus, there is a paucity of information on quality of care for persons affected by dementia. Large-scale population-based studies are needed to generate epidemiologic data on PCCS experiences and associated outcomes. That knowledge could be used to lobby for needed infrastructure and resources, a noted barrier of PCCS in our study. Two, most included studies captured views about PCCS through carers. Another literature review identified few studies that involved persons with dementia in planning their own care and support [68]. The lack of active and meaningful involvement of persons with dementia in shaping policies and interventions that impact their care and quality of life has been recognized as a problem [69, 70]. Therefore, future research on what constitutes PCCS for dementia must include those with dementia.

This review has many strengths. Use of a scoping review to combine findings from studies of various research designs has established a baseline of what is known about implementing PCCS in dementia and identified gaps in knowledge that must be addressed through further primary research. We employed rigorous scoping review methods [22, 23], and complied with standards for the conduct and reporting of scoping reviews and search strategies [24, 29]. The review was conducted by an international interdisciplinary team. By mapping studies to an existing PCC framework [25], we adapted that framework to the dementia context, and also elaborated it based on study findings. Several limitations must also be noted. Our search was limited to English language studies, so we may not have included relevant studies published in other languages. The search strategy may not have identified all relevant studies, or our screening criteria may have been too stringent. In particular, we defined PCCS in terms of interaction at the patient-carer-healthcare worker level, and included studies that referred to person-centred care or a synonymous term, which may have excluded potentially relevant studies. Included studies were few and conducted in few countries, therefore findings may not be broadly relevant and transferrable. While not required of scoping reviews [22, 23], included studies were not assessed for quality, calling for a cautious application in practice.

## Conclusions

Despite widespread advocacy, little prior research focused on PCCS for dementia in home, community or outpatient care. To address this knowledge gap, our scoping review of 22 studies published from 2001 to 2019 confirmed and elaborated 6 domains that constitute person-centred care and support (PCCS) for dementia, numerous approaches across those domains to achieve PCCS, and strategies to implement those approaches. We compiled this knowledge in a Framework of Person-Centred Care and Support for Dementia, which can be further refined through future research, and in the interim, employed by healthcare workers to plan or improve services. In-person inter-professional educational meetings improved perceived and observed healthcare worker knowledge about dementia and available support services; and knowledge about, attitude to and use of person-centred care approaches. We also identified numerous issues that warrant further investigation: evaluation of PCCS for dementia, enablers and barriers of PCCS among persons with dementia/carers, healthcare workers and organizations; strategies to implement PCCS approaches at the person/carer and organizational levels; and greater involvement of persons with dementia in defining PCCS. Future research must also explore PCCS needs and preferences by sex/gender and other intersectional factors such as ethnicity or culture to understand how to tailor PCCS.

## Supplementary Information

**Additional file 1.** Eligibility criteria

**Additional file 2.** MEDLINE search strategy

**Additional file 3.** Data extracted from included studies

## Data Availability

The dataset(s) supporting the conclusions of this article is(are) included within the article (and its additional file(s)).

## Abbreviations

PCC: Person-centred care
PCCS: Person-centred care and support
PICO: Population, Intervention, Comparisons, Outcomes
MeSH: Medical subject headings
