# Supplementary material for: How to implement person-centred care and support for dementia in outpatient and home/community settings: Scoping review
Source: BMC Health Serv Res. 2022 Apr 22;22:541. doi: 10.1186/s12913-022-07875-w (PMC9034625; doi:10.1186/s12913-022-07875-w)
Supplement: Supplementary file 1 — Additional file 1. Eligibility criteria [file 12913_2022_7875_MOESM1_ESM.docx]

Additional File 1. Eligibility criteria

ELIGIBLE

Population

- Persons aged 18+ of any sociodemographic characteristics with dementia or caring for a person with dementia (who may be referred to as spouses, partners, family, caregivers, informal caregivers or care partners)
- Practising healthcare professionals (e.g. physicians of any specialty, nurses, nursing assistants, personal support workers, physiotherapists, volunteers) who provide care to persons with dementia in any of the following settings (who may be referred to as caregivers or formal caregivers)
  - Outpatient care – refers to appointment-/office-based consultations/visits in hospital out-patient clinics or community-based primary care/family doctor offices
  - Home-based care – services and supports that help persons with dementia and carers to live and cope at home
  - Day centres – community settings providing venues for social interaction, entertainment or other support

Intervention/Issue

- An approach to care or support explicitly referred to as patient- or person-centred care or family-centred or a synonymous term that pertains to domains of McCormack’s PCC framework
- In prior research, we built on McCormack’s PCC framework to elaborate on these approaches

Framework of Patient-Centred Care

| PCC domains | Themes | Approaches |
| --- | --- | --- |
| Foster a healing relationship | Establish rapport | - Engage in friendly discussion prior to clinical discussion |
|  | Assume a non-judgmental attitude | - Maintain a neutral disposition - Speak in a respectful manner |
| Exchange information | Learn about context | - Allocate time to explore patient/caregiver context - Learn about lifestyle, social circumstances, personal goals, etc. |
|  | Allow time for discussion | - Avoid conveying a sense of rush to see the next person - Ask if there are remaining questions |
|  | Demonstrate active listening | - Sit facing the person and make eye contact - Record notes only after the person finishes speaking |
| Address concerns | Elicit emotions/concerns | - Take a holistic approach to care - Explicitly ask about feelings |
|  | Validate emotions/concerns | - Acknowledge hearing and understanding concerns - Reassure that it was appropriate to mention those feelings - Note that such feelings are normal or common |
| Manage uncertainty | Identify uncertainties or risks | - Explicitly note uncertainties about prognosis, and the risks and benefits of treatment options - Acknowledge the possibility of adverse outcomes |
|  | Discuss uncertainties or risks | - Describe the likelihood and nature of adverse outcomes - Answer questions about uncertainties or risks |
| Make decisions | Identify treatment or management options | - Note treatment or management options - Describe which are suitable or recommended, and why |
|  | Offer participatory decision-making | - Explore opinions or preferences about options - Invite them to make the final decision |
| Enable self-care | Describe the follow-up process | - Explain follow-up appointments (who to see, when) - Specify who to contact and how if concerns or issues arise |
|  | Offer self-care advice and instruction | - Provide verbal guidance on self-care and self-monitoring - Provide or offer referral to educational material or seminars |

Comparisons

- Explores or describes what patients/carers or healthcare workers view as PCC approaches for providing outpatient or home care, services or supports to persons with dementia and carers
- Identifies factors influencing the use or impact of PCC approaches (enablers, barriers, challenges)
- Evaluates the impact of strategies (policies, programs, interventions, tools) targeted at patients/carers, healthcare workers or both to promote or support PCC approaches including measurement of impact either before (i.e. baseline), during and/or after (possibly multiple time points) exposure to a strategy, or compares such approaches
- Sub-analyses: sex/gender/plus (social determinants: age, culture/ethnicity, education, country, urban vs rural dwelling)

Study Design

- Empirical studies including explicit methods of data collection and analysis
- Any qualitative or quantitative or multiple/mixed methods
- Reviews (e.g. scoping, systematic) are not eligible but we will screen references for eligible primary studies
- Published in English language in any country

Outcomes

- Any reported by eligible studies
- Focused on: awareness, knowledge, practice or impact of PCC approaches, or determinants of use or impact of PCC approaches

**NOT ELIGIBLE**

- Participants are trainees
- Setting is institutional care, or context is palliative or end-of-life care
- Arbitrarily use PCC or synonymous term referring to clinical care or management
- Conclude that PCC is needed
- Examine strategies to improve patient/carer communication
- Focus is engaging patients/carers in research
- Protocols, abstracts, editorials, letters, commentaries, clinical case studies or clinical guidelines
- Systematic reviews were not eligible, but we screened the references of those on eligible topics
