# Supplementary material for: How to implement person-centred care and support for dementia in outpatient and home/community settings: Scoping review
Source: BMC Health Serv Res. 2022 Apr 22;22:541. doi: 10.1186/s12913-022-07875-w (PMC9034625; doi:10.1186/s12913-022-07875-w)
Supplement: Supplementary file 2 — Additional file 2. MEDLINE search strategy [file 12913_2022_7875_MOESM2_ESM.docx]

Additional File 1. MEDLINE search strategy

| **#** | **Search Statement** | **Results** |
| --- | --- | --- |
| 1 | dementia/ or alzheimer disease/ | 135782 |
| 2 | Patient-Centered Care/ | 19321 |
| 3 | (patient centered or patient-centered or patient centred or patient-centred).mp. | 35318 |
| 4 | (person centered or person-centered or person centred or person-centred).mp. | 5973 |
| 5 | (family centered or family-centered or family centred or family-centred).mp. | 4602 |
| 6 | (client centered or client-centered or client centred or client-centred).mp. | 1527 |
| 7 | or/2-6 | 44450 |
| 8 | 1 and 7 | 1004 |
| 9 | limit 8 to (english language and humans) | 935 |
| 10 | limit 9 to (comment or editorial or lecture or letter or news) | 36 |
| 11 | 9 not 10 | 899 |
