# Supplementary material for: How to implement person-centred care and support for dementia in outpatient and home/community settings: Scoping review
Source: BMC Health Serv Res. 2022 Apr 22;22:541. doi: 10.1186/s12913-022-07875-w (PMC9034625; doi:10.1186/s12913-022-07875-w)
Supplement: Supplementary file 3 — Additional file 3. Data extracted from included studies [file 12913_2022_7875_MOESM3_ESM.docx]

Additional File 3. Data extracted from included studies

| Study  Author/year  Country  Aim | Objective  Setting  Aspect of care  Disease stage | Research design  Participants  Women-specific  PCC theory/model | Results | | | |
| --- | --- | --- | --- | --- | --- | --- |
|  |  |  | Identify PCC approaches | Assess if/how PCC approaches used | Describe enablers or barriers of PCC approaches | Evaluate impact of strategies to implement PCC approaches |
| Berglund (32)  2019  Sweden  Evaluate impact | Evaluate an educational program aimed at healthcare workers on how to provide person-centred dementia home care  Home  Overall management (care and support)  Stage: not reported | Qualitative (focus groups)  42 participants: care assistants, registered nurses, physiotherapists, occupational therapists, care managers, two educators, plus managers (home care ofﬁcers, politicians, management directors)  Women: sub-analyses not reported  PCC model/theory:  None reported; described as partnership with patient and family and putting the person at the forefront | --- | --- | --- | Five themes emerged:  1/ Knowledge about dementia and care:  Knowledge about dementia and care models, what to do when faced with problems, greater confidence, how to facilitate individualized care, skill in employing conversational approaches, created pride in their work  2/ Relationship-building:  Importance of building a good relationship with patient and carer, resulted in gaining easier access to home  3/ Open, flexible approach: Importance of positive attitude, open mind, ability to sense patient’s mood and convey calm preserved patient’s personhood and made it easier to provide care  4/ Continuity, flexibility:  Understanding patient’s and carer’s needs, and modifying time required for care enabled quicker decisions about care, which supported carers and improved patient confidence and sense of security  5/ Perceived impacts:  Improved health and nutritional status, delay in institutionalization, healthcare worker satisfaction, healthcare workers more open and curious about patient thoughts and needs, patients and carers more engaged in decision-making, heath problems detected earlier, infection prevention, reduced use of primary and hospital care |
| Hancox (33)  2019  United Kingdom  Describe determinants | Explore enablers and barriers of adherence to a home-based activity/exercise program facilitated by interdisciplinary healthcare workers to identify how to better personalize its implementation  Home  Support  Stage: mild | Qualitative (exercise diaries + interviews)  20 persons: 4 women, 16 men; mean age 76.6 years, range 68–91 year; and 19 carers: 17 women, 2 men  Women: 1 women adhered to exercise with support from a daughter; 2 women whose carer was a male spouse did not adhere (carer information not reported for remaining women who did not adhere)  PCC model/theory:  None reported; referred to tailored to the needs of specific individuals and designed and delivered to promote optimal motivation and adherence; used Theoretical Domains Framework to map determinants | --- | --- | Enablers of adherence were: developing a daily routine, strong practical and emotional support from healthcare workers and their carer, use of memory aids, positive past experience of exercise, clear purpose for doing the exercises, and perceived or tangible benefits from doing the exercises. Low adherence largely due to: lack of purpose and lack of practical or emotional support from their carer to routinize exercises between healthcare worker visits | --- |
| Ihara (34)  2019  United States  Evaluate impact | Evaluate the impact of a person-centred music listening intervention on depression, aggressive behaviour, mood, agitation and social engagement for persons with dementia  Day centre  Support  Moderate | Before-after cohort study with control group (instruments + observation)  31 persons exposed: 19 women, 12 men; mean age 81.3 years, range 57-93 years  20 persons in control: 15 women, 5 men; mean age 83.8 years, range 68-94 years  Women: sub-analyses not reported  PCC model/theory:  Kitwood – emphasizes well-being and quality of life as defined by the person, values individuals and carers, and creates an environment that takes into account the person’s past life and current status | --- | --- | --- | Instruments  Scores were not significantly different within or between groups after the intervention or at 6 weeks for depression or aggressive behaviour  Observations  Mood (smiling, joy, alertness) increased during the intervention and joy was sustained after. With respect to agitation, participants appeared relaxed and calm during the intervention, but this was not sustained after. Social engagement (eye contact, eye movement, talking) increased and sleeping decreased during and directly after the intervention |
| Hung (35)  2019  Canada  Evaluate impact | Evaluate an educational program aimed at healthcare workers on how to provide person-centred dementia care  Both inpatient and outpatient  Overall management (care and support)  Stage: not reported | Multiple methods (survey + focus groups)  310 staff: 145 nurses, 150 care workers, 3 physicians,3 social workers, 2 dieticians, 1 physiotherapist, 1 occupational therapist, 1 music therapist, 2 recreation staff and 2 managers; 95% women, 50% age 45+  Women: sub-analyses not reported  PCC model/theory:  McCormack & McCance, 2017 relationship, structural support and supportive environment | --- | --- | --- | Survey (n=297)  95% said education was useful and applicable, would have a positive impact on their ability to provide dementia care and would recommend to a colleague. In particular, they valued communication techniques (e.g. validation, personal space, stop-and-go)  Focus groups (3 physicians, 6 nurses, 4 allied healthcare, 11 care staff):  1/ Changing attitudes  Shifted view of persons with dementia as aggressive to having more compassion and understanding through deeper insight on patient perspective, emotions and communication  2/ Changing practices  Recognized complexity of dementia, focused on valuing patients as persons rather than dementia symptoms to be managed  3/ Conditions for PCC  Systems-thinking, teamwork and leadership support needed to create supportive conditions |
| Hall (36)  2018  United Kingdom  Assess PCC | Assess if and how persons with dementia received person-centred physiotherapy  Both inpatient and outpatient  Support  Stage: not reported | Qualitative (interviews)  11 persons: 6 with dementia, 5 carers; 8 women 3 men; all aged 65+  Women: sub-analyses not reported  PCC model/theory:  Edvardsson et al 2008. Acknowledge personhood, share decisions, prioritize relationship, personalize care | --- | 1/ Support to help engage and enable exercises, and build confidence  Physiotherapists di not look beyond dementia by taking time to get to know the person to tailor exercises due to perceived lack of knowledge, resources and time  2/ Shared decisions  Physiotherapists did not have an open discussion about how to adapt their treatment to overcome any difficulties due to dementia (e.g. short, regular treatments to create a routine)  3/ Abandonment  Lack of communication led to confusion, unclear expectations, and lack of clarity on why physiotherapy ceased | --- | --- |
| Jennings (37)  2018  United States  Identify PCC approaches | To explore patient and carer views of what constitutes patient-centred care  Home  Support  Stage: mild | Qualitative (focus groups)  43 participants:  7 persons with dementia: 2 women, 5 men; mean age 81 years (range 73-92 years)  36 carers: 26 women, 10 men; mean age 63 years (range 42-85 years); most were spouses or children; 15 Spanish speaking  Women: sub-analyses not reported  PCC model/theory:  None reported; refer to social and family relationships, functional independence, safety and physical well-being, support for direct care needs and day-to-day functioning, obtaining dementia-related services, medical care, emotional well-being, autonomy and the importance of meaningful activity | identified 41 goals for dementia care within five domains: medical care, physical quality of life, social and emotional quality of life, access to services and supports, and caregiver support  Caregiver goals included ensuring the safety of the person with dementia and managing caregiving stress. Participants with early-stage dementia identified engaging in meaningful activity (e.g., work, family functions) and not being a burden on family near the end of life | --- | --- | --- |
| Chung (38)  2017  United Kingdom  Identify PCC approaches | Explore how family carers support PCC to generate insight on how healthcare workers can better partner with carers  Home  Overall management (care and support)  Stage: not reported | Qualitative (interviews, focus groups)  Interviews  15 carers: 11 women, 4 men; 13 spouses, 2 daughters; aged 50-80 years  Focus groups  21 carers: 13 women, 8 men; aged 40-90 years  Women: sub-analyses not reported  PCC theory/model:  Kitwood personhood or agency: recognize past experience, personality, current strengths, abilities,  psychosocial needs and preferences | Carers encouraged autonomy and control by: being non-judgemental, promoting a sense of worth, maintaining stability and continuity in daily life; enhancing a sense of connection with their relative’s role and  identity using enjoyable activities; preventing inactivity; and attending to verbal and non-verbal behaviours to understand needs and preferences  They practiced constant decision-making for numerous activities elated to self-management, household management and entertainment, which required setting goals, gauging performance, making adjustments, and negotiation  This was time-consuming and demanding, often in isolation with no input form other family or professionals, so they lived with a lot of uncertainty and trial-and-error | --- | --- | --- |
| Guan (39)  2017  United States  Assess PCC  **same group as Lerner 2014 but different participants | Assess if and how disclosure of genetic risk influenced person-centred communication with patients and carers  Outpatient    Care (prognosis)  Mild cognitive impairment | Randomized trial (analysis of audio-recorded sessions)  79 patients: 35 women, 44 men; mean age 75.7, 96.2% Caucasian  Accompanying carer: 56 women, 23 men; mean age 68.0  Genotype results n=54; control n=25  Women: sub-analyses not reported  PCC theory/model:  None reported; described as opportunity to speak during interactions, and addressing psychosocial and emotional issues; Roter Interaction Analysis System codes medical visit communication as biomedical, lifestyle, psychosocial, etc. as well as positive (interest, warmth, empathy, respect) and negative (dominance, rushed) affect | --- | Genetic counselor contributed 63% of statements, while patients and companions contributed 19% and 18% respectively  Overall, 57% of genetic counselor statements responded to psychosocial and emotional issues, most often about lifestyle information, and partnership facilitation (checking understanding) and less often to express empathy or reassurance in response to patient emotion  Genetic counselors were more person-centred (provided more psychosocial and lifestyle information, used  more facilitative statements to clarify information or check  understanding) among controls (1.4 versus 1.0, p<0.001) | --- | --- |
| Wang (40)  2017  China  Evaluate impact | Evaluate the impact of a train-the-trainer education a program for primary care professionals on dementia knowledge and attitudes, and person-centred care delivery  Outpatient  Care (diagnosis, management)  Mild, moderate | Randomized controlled trial + focus groups  170 participants: 141 women, 29 men; mean age 30.9 years; 102 physicians, 68 nurses  30 took part in focus groups (characteristics not reported)  Women: sub-analyses not reported  PCC theory/model: not reported; referred to enable people with dementia to function  at the highest level of their capacity as long as possible and to relieve caregiver  burden | --- | --- | --- | Knowledge increased in intervention group compared with control at post-test (+3.86 adjusted  mean scores, 95% CI 2.98–4.74, p<0.001) and 3-month follow-up ((+4.53  adjusted mean scores, 95% CI 3.65–5.41, p<0.001)  Significant education effect on improved “Heartfelt” – increased mean score at post-test (1.19 adjusted mean scores, 95% CI 1.23-2.23, p<0.001), significant education effect on improved “Heartsink” – decreased mean score at post-test (-2.15 adjusted mean scores, 95% CI -2.87-1.44, p<0.001)  Attitude to dementia improved in intervention compared with control at post-test (+1.19 adjusted mean scores, 95% CI 1.23-2.23, p<0.001) and 3-month follow-up (+1.73 adjusted mean scores, 95% CI 1.23–2.23, p<0.001)  Person-centred care approach improved in intervention compared with control at post-test (+4.46 adjusted mean score, 95% CI 3.56-5.36, p<0.001) and 3-month follow-up (+2.82 adjusted mean scores, 95% CI 1.93–3.72, p<0.001)  Focus Groups:  Positive impact on dementia screening, referral to memory clinics, enhanced team collaboration, realized importance of care over importance of only medicine, more personalized plans for patients, developed information handouts for patients |
| Johansson (41)  2017  Sweden  Identify PCC approaches | Explore how to provide person-centred meal support for persons with dementia  Home  Support  Stage: not reported | Qualitative (focus groups)  22 persons aged 21-62 including nurses, nursing assistants, social workers and occupational therapists    Women: sub-analyses not reported  PCC theory/model: not reported | Overall: meals should be tailored to individual needs and preferences to foster independence  Understand cause of problems and needs of person and carer, engage both in making decisions  Enable independence by involving the person in planning, shopping or preparing, assisting but not taking over, if possible, and if not, helping to access meals at day centres or restaurants  Preserve routines on when and where to eat, create pleasant atmosphere, and include a social dimension | --- | --- | --- |
| Han (42)  2016  United States  Evaluate impact | Evaluate how a social visit program offers person-centred support  Home  Support (first year medical students make home visits for social interaction)  Stage: not reported | Qualitative (interviews)  5 carers: 3 women, 2 men aged 64-89 years  Women: sub-analyses not reported  PCC model/theory:  None reported; described as recognizing the whole person as having a unique history, needs, preferences and abilities | --- | --- | --- | Benefits for carers:  Knowing spouse enjoyed program, for some provided respite for personal time, for other enjoyed joint leisure time  Benefit for person with dementia:  Outlet to socialize with someone other than family, social and intellectual stimulus |
| Gaugler (43)  2015  United States  Evaluate impact | Evaluate the impact of an online educational program on carer knowledge of person-centred approaches  Home  Overall management (care and support)  Stage: not reported | Before-after cohort (survey)  41 carers: 37 women, 4 men; mean age 58.0 years, range 39-89 years  Women: sub-analyses not reported  PCC model/theory:  Edvardsson 2008 described as support the rights, values, and beliefs of the individual; involve  them and provide unconditional  positive regard; enter their world and assumes that there is meaning in all behaviour, maximizes their potential and shares decision-making | --- | --- | --- | Survey:  Knowledge increased significantly  13.07 (SD = 2.82) correct responses (t = 6.60, df = 40, p < 0.001)  Carers said they had greater confidence and mastery in their caregiving skills and communication. Aspects deemed particularly positive  included the comprehensive  content, the use of real individuals with dementia and their caregivers in video care vignettes, and the flexibility of online delivery |
| Edwards (44)  2015  United Kingdom  Evaluate impact | Evaluate the impact of an educational intervention to promote person-centred primary care for persons with cognitive decline  Outpatient  Care (diagnosis)  Mild cognitive impairment | Before and after cohort (survey)  94 participants: 30 physicians, 21 nurses or nursing assistants, and 43 clerical staff  Women: participant characteristics and sub-analyses not reported  PCC model/theory:  Based on Kitwood; described as takes a holistic approach that emphasises the perspective of the person with dementia and their self-defined experiences and needs | --- | --- | --- | Several measures increased significantly for non-physicians:  Nursing and other staff may be well-placed to spot decline (66% to 89%, p=0.002); If disoriented, I would further question them or invite them to another appointment (58% to 92%, p=0.00001); Behavioural and functional problems are symptoms as much as memory loss is (65% to 92%, p=0.0002); Support services exist (58% to 92%, p=0.00001)  The only item to increase significantly for both groups was good understanding of what constitutes PCC: physicians (35% to 82%, p=0.0002), non-physicians (10% to 60%, p=0.0000001) |
| Smythe (45)  2015  United Kingdom  Identify PCC approaches  Describe determinants | Identify PCC approaches among carers and healthcare workers; Explore determinants of person-centred dementia care among healthcare workers  Inpatient and outpatient  Overall management (care and support)  Mild, moderate | Qualitative (focus groups)  70 healthcare workers: nurses, nursing assistants, occupational therapists and physiotherapists  16 carers  Women: characteristics and sub-analyses not reported  PCC model/theory:  Kitwood - emphasise individual identity, personal history and social relationships in addition to neurological and health factors | Healthcare workers:  Most felt that PCC skills were inherent in their roles. Qualities essential to delivery of PCC: attributing value to people with dementia, respect for subjective experiences, a non-judgemental approach, patience, an emphasis on seeing the person as a whole, enabling and empowering the individual with dementia and taking a person-centred approach  Carers  Emphasized three qualities they believed were innate rather than coming from training: (1) having a good heart and a caring nature; (2) using a person-centred approach defined as ability to listen and understand, create a warm atmosphere, and demonstrating respect and empathy; and (3) kindness and patience  Caring nature, kindness, and patience is needed from professionals when caring for people with dementia | --- | Barriers:  Lack of time, inadequate staffing, pressured environment, lack of control and perceived low status within organization  Enablers:  Mutual support from colleagues, job satisfaction, connections with individuals with dementia, experiential learning | --- |
| Edwards (46)  2014  United Kingdom  Identify PCC approaches | Explore what constitutes person-centred primary care for the diagnosis of dementia  Outpatient  Care (diagnosis)  Mild | Qualitative (focus groups)  Patients/Carers:  3 persons with dementia and 4 carers  Healthcare workers:  10 individuals – 1 general practitioner, 1 general nurse, 1 psychiatric nurse and 7 allied health care professionals  Women: characteristics and sub-analyses not reported  PCC model/theory:  Kitwood - holistic  approach that emphasises the perspective of the person with dementia and their self-defined  experiences and needs | Reframing dementia as cognitive decline:  Allows person and family to adjust to the diagnosis, and allows physician to identify and refer those who might benefit from specialist services  Triggers for recognizing dementia:  In addition to or instead of memory loss, other signs included behaviours and challenges in daily activities  Engaging the entire primary care team:  Users and carers often felt rushed by the physician, but other staff are well-placed to recognized decline, and they can raise issue with physician  Link between primary and secondary care:  Physicians would benefit from knowing about available care and support services | --- | --- | --- |
| Lerner (47)  2014  United States  Assess PCC  **same research team as Guan 2017 but different participants | Assess if and how genetic counseling sessions were person-centred  Outpatient  Care (diagnosis)  Mild | Randomized trial (analysis of audio-recorded sessions)  262 participants:  183 women, 79 men; mean age 58.3 years (range 33-86 years); 212 Caucasian  Women: No significant differences by age, gender or race  PCC model/theory:  Mead and Bower - bio-psychosocial perspective, sharing of power and responsibility  between patient and provider, and the development of a  therapeutic alliance in which both parties develop common goals and a personal bond. Also used Roter Interaction Analysis System codes medical visit communication as biomedical, lifestyle, psychosocial | --- | Pattern analysis identified three communication categories:  Biomedical provider driven (40% of sessions); biomedical patient driven (34.4%); and psychosocial patient driven (25.6%)  During psychosocial sessions, providers gave less biomedical information, asked more psychosocial questions, and made more efforts to  build partnership than other sessions. Participants in PPC  sessions shared more psychosocial information than participants in other patterns. There were also a greater proportion of utterances by participants compared with the others groups | --- | --- |
| McClendon (48)  2013  United States  Identify PCC approaches | Explore what constitutes person-centred approaches to care by informal caregivers  Home  Support  Stage: not reported | Survey (used multiple existing instruments)  148 carers:  62% women; 91% Caucasian; mean age 69 years  Women: women caregivers more likely to provide PCC compared with men  PCC model/theory:  Kitwood - promotion of personhood based on autonomy, individuality, dignity,  and environmental support | Personalized care:  promote meaningful  activities that reflect individuality and facilitate autonomy  Compensatory caregiving: environmentally and personally compensate for the  impairments of the person with dementia  Respectful caregiving:  Respect capabilities and limitations by not reacting in a judgmental way  Predictors included:  Greater carer neuroticism, openness, conscientiousness, agreeableness | --- | --- | --- |
| Kirkley (49)  2011  United Kingdom  Describe determinants | To describe enablers and barriers of implementing person-centred care, particularly factors related to organizational culture  Day care services  Support  Stage: not reported | Qualitative (interviews, focus groups)  70 participants:  52 women, 18 men; 44 strategic or operations managers, 17 frontline staff; organizations largely provided support in home or community  Women: sub-analyses not reported  PCC model/theory:  Hughes et al. 2008  respect for individual, meaning, therapeutic alliance, social context and relationships, holistic health and well-being, expert lay knowledge, shared responsibility, communication, autonomy, professional as person | --- | --- | Varied understanding of PCC  How organisational  culture thought to influence PCC: understandings  of PCC, attitudes to service development,  service priorities, valuing staff, solution-focused approaches  Barriers  Resource constraints and the knowledge,  attitudes and personal qualities of staff  Enablers  Leadership style, how  managers support and value staff, risk management, opinion leaders, policy documents | --- |
| Robinson (50)  2010  United Kingdom  Evaluate impact | Evaluate the impact of an educational intervention to promote person-centred care by old age psychiatrists  Outpatient  Overall management (care and support)  Stage: not reported | Before-after cohort (survey)  40 psychiatrists:  20 women, 20 men; 21 general and 10 geriatric psychiatrists, 30 10+ years in practice  Women: sub-analyses not reported  PCC model/theory:  Hughes 2008 review of “centredness” revealed 9 domains in three themes: interpersonal relationship, patient involvement, holistic approach | --- | --- | --- | Three months after the workshop, 59% had made one or more changes to the structure of consultations,  71% had used new communication skills and 56% had reflected further on their practice. Overall, participants reported mean 6.3 changes; more often these were skill changes (45% were implemented) compared with consultation structure (29% implemented) or reflection (20% implemented) |
| Vernooji-Dassen (51)  2010  The Netherlands  Describe determinants | Explore enablers and barriers of person-centred family counseling  Outpatient  Support  Stage: not reported | Qualitative (interviews)  3 long-time counsellors  All women; one each in 60s, 80s and 90s; 2 social workers, 1 gerontologist  Women: all views expressed by women; no sub-analyses of carer/family dynamics reported  PCC model/theory:  None reported; described as improving quality of life and maximizing function in the context of existing deficits | --- | --- | Barriers:  When counsellors were confronted with reluctance to be helped and with provocations to get involved in family conflicts; children who live at great distance from their parents, feeling like an unwanted intruder  Enablers:  Being aware of problems within family system, maintaining a neutral position, following the family’s lead; illness of the parents was an opportunity to reconnect and rebuild a bridge to the family; counselling provided a safe environment to offer help in acceptable and creative ways | --- |
| Zaleta (52)  2010  United States  Assess PCC | Assess if disclosure of dementia diagnoses were person-centred  Outpatient  Care (diagnosis)  Mild | Cohort (analysis of audio-recorded sessions)  54 patients:  33 women, 21 men; mean age 74.1 (range 58-91 years), 51 Caucasian  54 carers:  38 women, 16 men; mean age 65.6 (range 43-86 years), 50 Caucasian  Women: physician PCC behaviour not associated with patient (r=-0.22, p>0.10) or caregiver (r=0.21, p>0.10) gender or patient (r=-0.21, p>0.10) or caregiver (r=0.12, p>0.90) age  PCC model/theory:  None reported; described as shift in balance from a physician-dominated interaction to one where physicians solicit patient perspectives and enhance patient feelings of partnership and understanding; also used Roter Interaction Analysis System codes medical visit communication as biomedical, lifestyle, psychosocial | --- | Physicians  engaged in more positive rapport building such as agreeing with patient or carer (mean 28.04, SD 17.55, p<0.001) and facilitation and patient activation such as checking for understanding (mean 23.22, SD 15.85, p<0.001) per session compared to emotional rapport building (mean 6.59, SD 5.63) such as reassurance or empathy | --- | --- |
| Ericson (53)  2001  Sweden  Identify PCC approaches | Explore family and healthcare worker views on what constitutes person-centred dementia care  Home  Support  Stage: not reported | Qualitative (interviews)  20 family carers:  13 women, 7 men (9 wives, 6 husbands, 3 daughters, 1 brother, 1 sister)  17 healthcare workers:  6 nurses, 6 nursing assistants, 2 health system managers, 1 occupational therapist, 2 day care managers  Women: sub-analyses not reported  PCC model/theory:  None reported; described as personhood – person treated with dignity and respect; focuses on abilities rather than deficits (Pickard 1999) | Family carers  Motivated to keep person in their own home for several reasons: familiar environment, family  helped the person to be more actively engagement and maintain dignity, better able to provide stimulating activity at home, intimate knowledge of person attuned to issues not apparent to others to optimize care and support  Healthcare workers  Considered home ideal environment, but homes suitable when needed, attention to individual needs important and optimized through continuity of one or a few paid carers so they get to know person and develop insight to their needs; conscious of need to support family carers and to work in partnership with family – saw key role as providing information about dementia and services available | --- | --- | --- |
